# Treatment of Patients with Mild to Moderate Ulcerative Colitis: A Middle East Expert Consensus

**DOI:** 10.3390/jcm12216929

**Published:** 2023-11-04

**Authors:** Sameer Al Awadhi, Mohamed Alboraie, Emad Aldin Albaba, Abdulelah Almutairdi, Monther Alsaad, Nahla Azzam, Husam Barakat, Ferdinando D’Amico, Silvio Danese, Mohamed El Kady, Hossam Ghoneim, Waseem Hamoudi, Ahmad Jazzar, Mahmoud Mosli, Hany Shehab, Awni Abu Sneineh

**Affiliations:** 1 Digestive Diseases Unit, Rashid Hospital, Dubai 003206, United Arab Emirates; 2 Department of Internal Medicine, Al-Azhar University, Cairo 11884, Egypt; alboraie@azhar.edu.eg; 3 Department of Medicine, Almana General Hospital, Alkhobar 31952, Saudi Arabia; emadalbaba@yahoo.com; 4 Department of Medicine, King Faisal Specialist Hospital and Research Center, Riyadh 11564, Saudi Arabia; aalmutairdi@kfshrc.edu.sa; 5 Al Madar Medical Centre, Sharjah P.O. Box 80789, United Arab Emirates; monthermd@yahoo.com; 6 Division of Gastroenterology, Department of Medicine, College of Medicine, King Saud University, Riyadh 11451, Saudi Arabia; nahla5_99@yahoo.com; 7 Department of Gastroenterology, Yarmouk University, Irbid 21163, Jordan; gastromd@outlook.com; 8 Gastroenterology and Endoscopy, IRCCS Ospedale San Raffaele and Vita-Salute San Raffaele University, 20132 Milan, Italy; damico.ferdinando@hsr.it (F.D.); danese.silvio@hsr.it (S.D.); 9 Department of Biomedical Sciences, Humanitas University, Pieve Emanuele, 20132 Milan, Italy; 10 Department of Medical Pharmacology, Faculty of Medicine, Cairo University, Cairo 11559, Egypt; dr.m.elkady@gmail.com; 11 Immunology and Allergy Department, Medical Research Institute, Alexandria University, Alexandria 5424041, Egypt; dr.hghoneim@gmail.com; 12 Internal Medicine Department, Al-Bashir Hospital, Amman 11151, Jordan; waseem6520012001@yahoo.com; 13 Gastroenterology Division, Sheikh Khalifa Medical City, Abu Dhabi 51900, United Arab Emirates; ahmad.jazzar@burjeeldaysurgery.com; 14 Department of Medicine, King Abdulaziz University Hospital, Jeddah 21589, Saudi Arabia; mmosli@kau.edu.sa; 15 Integrated Clinical and Research Center for Intestinal Disorders (ICRID), Gastroenterology Division, Endemic Medicine Department, Cairo University, Cairo 3725121, Egypt; h.shehab@kasralainy.edu.eg; 16 Gastroenterology and Hepatology, University of Jordan, Amman 11942, Jordan; aabusnei@hotmail.com

**Keywords:** 5-ASA, mesalazine, budesonide MMX, ulcerative colitis, inflammatory bowel disease

## Abstract

The prevalence of ulcerative colitis (UC) in the Middle East is increasing, impacting the economic and healthcare burden. The management of patients with mild to moderate UC is still a challenge as several factors can affect optimal care, including drug choice, induction and maintenance dose, treatment optimization and de-escalation, therapy duration, monitoring, and safety profile. We conducted an expert consensus to standardize the management of patients with mild to moderate UC. Sixteen experts in inflammatory bowel diseases, through a well-established and accepted Delphi methodology, voted and approved eight statements in order to provide practical guidance to clinicians in the Middle East.

## 1. Introduction

Ulcerative colitis (UC) is a chronic inflammatory bowel disease (IBD) with a remitting and relapsing course [1]. It has a significant burden on both patients’ quality of life and health care. The prevalence of UC has increased significantly in the last three decades and the United States of America represents the country with the highest prevalence rate (422 per 100,000 population) [2]. However, the UC prevalence is increasing globally, reaching rates of 106.2 per 100,000 people in the Middle East [3]. The emergence of this disease underlines the need to implement knowledge and tools for UC management in order to obtain an early diagnosis and to set an adequate treatment to achieve optimal disease control.

Typically, UC is stratified by disease activity into mild–moderate or moderate–severe [4]. Approximately 50% of patients with UC have a mild disease [5]. Different therapeutic algorithms are available for UC patients depending on disease activity [4]. The 5-aminosalicylates (5-ASA) are the first-line therapy for both inducing and maintaining disease remission in mild to moderate UC. Unfortunately, a considerable percentage of patients treated with 5-ASA fail to respond to treatment or lose response over time [6]. Several variables could affect the efficacy of the therapy including drug choice and dosage, administration route (oral or rectal), duration, optimization, and safety profile. An expert consensus meeting was conducted to overcome these limitations, standardize the management of patients with mild to moderate UC, and provide practical guidance to clinicians in the Middle East.

## 2. Materials and Methods

A literature review based on PRISMA guidelines was conducted by two authors (FD and SD) to investigate all studies involving mesalazine and budesonide MMX in patients with mild to moderate ulcerative colitis. PubMed, Embase and Web of Science databases were used for research. The following search terms were used: “mesalazine”, “5-ASA”, “aminosalicylates”, “budesonide MMX”, “cortiment”, “ulcerative colitis”, “UC”, inflammatory bowel disease”, and “IBD”. Based on the available evidence, eight statements were preliminarily created (Supplementary Materials Table S1). These statements were then discussed and voted upon by 16 experts (from Egypt, Italy, Jordan, Saudi Arabia, and United Arab Emirates) in an in-person international meeting. Only physicians following more than 1000 IBD patients per year were invited to participate. The meeting took place in Dubai on 20 May 2023. The voting was conducted using a polling system. A Delphi methodology was applied. Two rounds of voting were planned. To be accepted, at least 75% of the participants had to vote in favor of the statement. If the agreement threshold was not met after the first round of voting, the statement was further discussed, amended, and voted on again in a second round. If no agreement was reached even after the second round, the statement was excluded from the final list. Throughout the process, experts were also allowed to propose new statements, which were then discussed and voted on using the same methods. After the meeting, all the experts were involved in drafting the manuscript, which was subsequently approved in its final version by all the authors.

## 3. Results

Seven statements (7/8, 87.5%) were approved after the first round of voting (Table 1). One statement was rejected (1/8, 12.5%). A statement was proposed during the meeting and approved on the first attempt.

**Statement 1.**  *We suggest the use of oral 5-ASA (from 2.0 to 4.8 g/day) combined with topical 5-aminosalicylates for 8 weeks to induce remission in patients with mild to moderate proctitis (suppository 1 g/day) or left-sided ulcerative colitis/pancolitis (enema ≥ 1 g/day) (agreement 16/16, 100%).*

The 5-ASA is the gold standard for the induction of remission in patients with mild to moderate UC according to multiple international guidelines [4,7,8,9]. The efficacy of mesalazine in this setting is proven by several meta-analyses [10,11,12,13,14,15,16]. There is also evidence that the combination of oral and topical mesalazine is more effective in inducing disease remission than oral therapy alone [10,11,17,18]. However, there is no agreement regarding the type of 5-ASA to be preferred and its optimal dosage. A Cochrane meta-analysis found no difference in inducing remission between different formulations of oral 5-ASA [15]. Conversely, a randomized clinical trial compared the efficacy and safety of high (4.8 g/day) and low (2.4 g/day) doses of oral 5-ASA in patients with mild to moderate UC [19]. After 6 weeks of treatment, the clinical remission rate, defined as stool frequency normalization and absence of rectal bleeding, was statistically higher in patients receiving the highest dose (43% vs. 35%, *p* = 0.04). Similarly, another randomized clinical trial investigated the rate of UC patients achieving endoscopic remission (defined as endoscopic Mayo score ≤ 1) after 6 weeks of therapy with 5-ASA 2.4 g per day or 4.8 g per day [20]. Interestingly, patients treated with the higher dose achieved endoscopic remission more frequently than those treated with the lower dose (80% vs. 68%, *p* = 0.012). Regarding rectal therapy, a randomized clinical trial by Campieri and colleagues compared the efficacy of three different formulations of 5-ASA enema (1 g, 2 g, and 4 g per day) [21]. No differences were found between study arms, suggesting that the lowest dose of a 1 g 5-ASA enema was sufficient for symptom control in subjects with left-sided UC/pancolitis. Instead, 5-ASA suppositories of 1 g per day are an effective therapeutic option in proctitis and are preferred by patients over the 500 mg formulations, which are applied more times a day [22,23]. The choice of 5-ASA dosage to induce remission should take into consideration several factors such as patient preference, disease location, and severity. A 5-ASA dosage ≥ 2 g per day might be preferred in proctitis, while in more extensive diseases a higher dosage (≥4 g) should be considered. In patients with a slightly above normal number of bowel movements and rare rectal bleeding, the lowest drug dose (≥2 g per day) could be considered a good compromise in the risk/benefit ratio of the therapy. On the other hand, in those with a moderate activity of disease (based on partial Mayo score), the highest dosage (≥4 g per day) should be preferred.

**Statement 2.**  *We recommend optimizing oral 5-aminosalicylates (≥4 g/day) in patients with mild to moderate ulcerative colitis who do not respond to therapy with oral 5-aminosalicylates at a dose < 4 g/day or experience clinical, biochemical, or endoscopic recurrence of disease (agreement 16/16, 100%).*

In the last decade, treatment targets of UC have evolved from symptom control to endoscopic remission [24]. Furthermore, fecal calprotectin (FC) plays an increasingly important role in the management of UC being recognized as an intermediate therapeutic target [25]. For this reason, medical therapy should be escalated if these goals are not met. A randomized clinical trial by Osterman and colleagues investigated the effect of 5-ASA therapy escalation in patients in clinical remission with an increase in FC levels (>50 µg/g) [26]. The patients were randomized into two groups: the first continued the ongoing therapy, and the second increased the drug dosage by 2.4 g per day. Patients in the experimental arm had higher rates of clinical and biochemical remission (FC < 50 µg/g) than the control group (26.9% vs. 3.8%, *p* = 0.0496). Furthermore, patients with FC values ≥200 µg/g experienced earlier clinical disease recurrence than those with FC < 200 µg/g, emphasizing the important predictive role of FC in UC. A phase III parallel-dosing study compared the efficacy of the induction regimen with 5-ASA 4 g/day versus 5-ASA 2.25 g/day in patients with UC [27]. After 8 weeks, patients treated with 5-ASA 4 g/day had a significantly higher efficacy rate compared to the control group (76.3 vs. 45.8%, *p* = 0.001) without differences in terms of safety. A decision tree model derived from a meta-analysis including 10,000 newly diagnosed patients with mild-to-moderate UC investigated the outcomes of patients undergoing 5-ASA optimization [28]. A group of patients was treated with a low-dose 5-ASA (2–2.9 g per day) without the possibility of optimizing the dosage (5-ASA > 3 g per day), while another group was treated with an optimized 5-ASA. In the optimized group, a relative increase in achieving remission of 39% was detected. Moreover, there was a reduction in the use of systemic steroids and biological drugs, a lower rate of adverse events and a cost saving of £354 per patient compared to the standard group. Another study conducted in the United Kingdom showed that patients treated for 12 weeks with high doses of 5-ASA (4.8 g per day) had a 10% reduced risk of experiencing hospitalization or surgery compared with the low-dose group (2.4 g per day), confirming the cost-effectiveness of this strategy [29]. This evidence suggests that optimizing 5-ASA is effective in patients who lose the response after low-dose 5-ASA treatment, allowing them to not only achieve remission again, but also reducing the risk of complications positively impacting on indirect cost savings. For this reason, patients not achieving treatment targets according to STRIDE II recommendations should undergo an escalation of medical therapy [25]. In patients with distal colitis who do not respond to 5-ASA therapy, topical steroids may be considered to induce disease remission [4].

**Statement 3.**  *5-aminosalicylates at a dose ≥4 g/day are not associated with an increased risk of adverse events or nephrological damage compared with 5-aminosalicylates at a dose ≤2 g/day (agreement 16/16, 100%).*

The use of 5-ASA has been associated with nephrotoxicity ranging from indolent forms to end-stage kidney failure [30,31]. The mechanism underlying renal damage is not known, but several studies have addressed this important issue. A randomized placebo-controlled trial evaluated the safety of oral 5-ASA in patients with UC by stratifying it according to drug dosage (1 g, 2 g, or 4 g per day) [32]. No differences between 5-ASA and placebo were identified. Furthermore, higher doses were not associated with an increased risk of side effects. A systematic review of randomized clinical trials investigated the risk of renal adverse events in patients treated with 5-ASA by comparing high vs. low dosing [33]. Importantly, no differences between the two groups were identified. A large British epidemiological study including about 40,000 patients evaluated the incidence of renal disease in IBD patients treated with 5-ASA [34]. The outcomes of 5-ASA-treated IBD patients were compared to an IBD cohort not undergoing 5-ASA therapy and a non-IBD control cohort not receiving 5-ASA. The incidence of renal disease in the IBD patients treated with 5-ASA was 0.17 cases per 100 patients per year and was similar to the other cohorts (0.08 and 0.25 cases per 100 patients per year, respectively). In addition, there was no difference in renal impairment when stratified by dose or type of 5-ASA, suggesting that the pathophysiological mechanism underlying renal disease is idiosyncratic and not dose-related. Although rare, it is crucial to monitor kidney function to prevent the risk of irreversible complications. To date, there are no globally accepted guidelines for the assessment and monitoring of renal function [35,36]. However, serum creatinine, estimated glomerular filtration rate (eGFR), and 24 h proteinuria are the most frequently used tests [30]. As far as the timing of monitoring is concerned, an assessment of renal function should be performed before starting therapy with 5-ASA and then should be evaluated more frequently during the first year of therapy (three to four times per year) and then one to two times a year thereafter. Of note, the patient’s comorbidities and any concomitant renal disorders should be taken into consideration, setting up personalized and closer monitoring.

**Statement 4.**  *In case of loss of response upon de-escalation of medical therapy, 5-aminosalicylates should be re-escalated (≥4 g/day) and maintained at a stable dosage (agreement 14/16, 87.5%).*

There is evidence that patients who achieve remission on high doses of 5-ASA may experience a disease recurrence upon de-escalation of the therapy. A prospective Japanese study investigated whether therapy optimization is safe and effective in patients with UC who relapse under a low-dose (1.5–2.25 g/day) 5-ASA maintenance therapy [37]. The patients were treated with oral mesalazine at 4.0 g/day. After 8 weeks, two thirds of the subjects achieved clinical improvement, about half of them were in clinical remission and one quarter was in endoscopic remission. No side effects occurred after 5-ASA optimization and the therapy was well tolerated. A phase 4 study, the Momentum trial, evaluated the efficacy of mesalazine 2.4 g once daily as a maintenance therapy in patients who achieved remission after 8 weeks of 4.8 g/day of mesalazine [38]. Importantly, approximately half of the patients who were in remission at the end of the induction phase were still in remission after 1 year of low-dose 5-ASA maintenance therapy, suggesting that many patients were undertreated. A non-interventional Dutch study explored whether the dose and duration of 5-ASA have an impact on the UC outcomes [39]. Interestingly, patients treated with 5-AS >4 g/day for a long time had a lower risk of relapse compared to those undergoing 5-ASA 2-<4 g/day (26.6% vs. 62.5%, *p* = 0.04). Furthermore, data from a retrospective study confirmed that long-term high doses of 5-ASA were associated with a reduced risk of recurrence compared to short-term therapy (29.8% vs. 48.3%, *p* < 0.05) [40]. The impact of 5-ASA optimization on outcomes of UC patients was also evaluated in a randomized clinical trial comparing two different doses of 5-ASA (2.4 g per day or 4.8 g per day) [41]. Only patients in clinical, endoscopic, and histological remission were eligible for the study. In addition, a history of frequent disease recurrences (at least 3 per year) was required. The patients treated with high doses of 5-ASA had a higher remission rate at 12 months than the control group (87.5% vs. 69.2%, *p* = 0.03). No differences in terms of compliance or adverse events were found supporting the use of high-dose 5-ASA as a maintenance therapy in the patients with a history of multiple relapses. Therefore, patients experiencing a loss of response according to the STRIDE II recommendations upon de-escalation of medical therapy should be re-escalated to maintain an optimal disease control.

**Statement 5.**  *In patients with mild to moderate ulcerative colitis who are primarily unresponsive or losing response to an optimized dose of 5-aminosalicylates, budesonide MMX (9 mg/day) could be considered as add-on therapy for 8 weeks to induce disease remission (agreement 16/16, 100%).*

Budesonide MMX is a corticosteroid with a limited systemic effect as it has a first-pass hepatic metabolism [42]. The efficacy of budesonide MMX was tested in a randomized placebo-controlled clinical trial [43]. Patients with mild to moderate UC were treated with two different doses of budesonide (9 and 6 mg/day), mesalazine 2.4 g/day or placebo for 8 weeks. Patients treated with budesonide 9 mg achieved clinical remission at a significantly higher rate than 5-ASA and placebo groups (17.9% vs. 12.1% and 7.4%, *p* < 0.05 for both comparisons). There was no difference between study arms in the rate of adverse events and serious adverse events, underlining the safety profile of budesonide MMX. These data were confirmed by another randomized study including patients treated for 8 weeks with two different doses of budesonide (9 mg and 6 mg), a controlled 9 mg of ileal-release budesonide, or a placebo [44]. Overall, 9 mg of budesonide MMX was more effective than the placebo and ileal-release budesonide in achieving the primary endpoint of combined endoscopic and clinical remission (17.4% vs. 4.5% and 12.6%; only the comparison with placebo was statistically significant with *p* = 0.0047). Again, the safety profile of budesonide MMX was reassuring, as there was no difference between study arms. Moreover, there is evidence that budesonide MMX is also effective in combination with 5-ASA, when the latter does not allow for optimal disease control. In a randomized clinical trial by Rubin and colleagues, patients with mild to moderately active UC despite 5-ASA therapy (dosage > 2.4 g per day) were randomized to receive 9 mg of budesonide MMX or a placebo in addition to 5-ASA for 8 weeks [45]. Patients treated with budesonide MMX were more likely to achieve a combined clinical and endoscopic remission than the placebo group (13.0% vs. 7.5%, *p* = 0.049). In addition, histological remission was also achieved by more patients treated with budesonide MMX than with placebo (27.0% vs. 17.5%; *p* = 0.02). There was no increase in the rate of side effects in patients treated with either therapy, thus supporting this option in 5-ASA refractory patients. The efficacy and safety of budesonide MMX as an add-on therapy were also evaluated in a real-life setting. In a prospective, multicenter, international study, patients with mild to moderate UC treated with high-dose 5-ASA were divided into three groups: one group added 9 mg of budesonide MMX 14 days after 5-ASA optimization, one group added 9 mg of budesonide MMX within 14 days from 5-ASA optimization, and one group was treated with 9 mg of budesonide MMX monotherapy [46]. Approximately 60% of patients had clinical improvement after the therapy with budesonide MMX. Surprisingly, clinical improvement was greater in patients treated with budesonide MMX as an add-on to 5-ASA (64.3% and 62.1%, vs. 33.3%, *p* = 0.0096).

**Statement 6.**  *If there is no clinical response to therapy within 4 weeks, medical therapy should be escalated in patients with mild to moderate ulcerative colitis (agreement 15/16, 93.7%).*

The timing for assessing the response to treatment is hotly debated. A post hoc analysis of two randomized clinical trials showed that patients treated with 5-ASA experienced clinical improvement as early as 2 weeks [47]. Patients who responded early to therapy were more likely to maintain the response. However, it should be emphasized that when disease recurrence occurs, a differential diagnosis is mandatory [48]. Therefore, infections and other causes of symptoms must be investigated and ruled out before making treatment decisions. A randomized clinical trial demonstrated that approximately 90% of patients treated with the combination of high-dose oral 5-ASA and rectal 5-ASA achieved clinical improvement after 4 weeks and almost half were in remission [18]. Similarly, another trial showed that the majority of subjects undergoing a combination therapy with oral and rectal 5-ASA achieved mucosal healing and a significant improvement in their health-related quality of life after 4 weeks [17]. A pooled analysis of five trials including over 1000 UC patients confirmed that resolution of stool frequency and rectal bleeding occurred on average after 4 and 2 weeks, respectively, from the start of 5-ASA therapy [49]. Additionally, two-thirds of those who achieved clinical remission during the induction phase maintained remission after 12 months. A time of 4 weeks could represent a fair compromise to evaluate the response to the drug, exclude confounding factors, and eventually change the therapy.

**Statement 7.**  *Oral 5-aminosalicylates at a dose ≥ 2 g/day are recommended to maintain disease remission and prevent colorectal cancer risk in mild to moderate ulcerative colitis (agreement 16/16, 100%).*

UC is a chronic disease that requires long-term therapy to maintain remission and prevent the risk of recurrence [4]. A randomized clinical trial enrolling 101 patients with UC showed that patients treated with 5-ASA 2 g daily had a significantly reduced risk of recurrence compared to patients treated with a placebo [50]. Its well-established safety profile and low cost support the use of 5-ASA to ensure optimal disease control. In addition, the long-term use of 5-ASA has been associated with an oncoprophylactic effect due to a significant reduction in transcript levels of colorectal carcinogenesis genes [51]. A nationwide, retrospective study including more than 1000 patients with IBD investigated the role of mesalazine on the risk of advanced-stage IBD-associated intestinal neoplasia [52]. Overall, 5-ASA use was associated with a significant reduction in the risk of advanced cancer in UC (OR = 0.628, 95% CI, 0.401–0.982, *p* = 0.041) supporting its role in colorectal cancer prophylaxis. A systematic review and meta-analysis of observational studies investigated the risk factors for advanced colorectal cancer (defined as high-grade dysplasia or colorectal cancer) in patients with IBD [53]. Overall, 5-ASA was identified as a protective factor against the onset of advanced colorectal cancer in both uni- and multi-variate analyses (pooled odds ratio, 0.51; 95% confidence interval, 0.39–0.66) with a moderate degree of evidence.

**Statement 8.**  *Oral 5-aminosalicylates should be administered once a day to improve adherence in patients with ulcerative colitis (agreement 16/16, 100%).*

Patient adherence to therapy is one of the main challenges associated with chronic oral medications. Several factors influence treatment adherence including the administration route and frequency, education level, and patient preference [54]. Numerous studies have shown that there is no difference in efficacy and safety between patients taking once-daily 5-ASA compared with those taking a split dose [54,55,56,57,58,59]. However, the once-daily dose is preferred by patients, is associated with a higher rate of self-reported adherence and does not lead to an increased rate of side effects compared to fractionated therapy [54,55,60]. For this reason, daily dosing should be preferred and recommended by clinicians in their clinical practice.

## 4. Research Gaps and Study Limitations

Although the management of patients with mild to moderate UC is based on consolidated scientific evidence, there are still gaps in our knowledge. Firstly, there is no commonly accepted definition of mild to moderate UC [61]. This contributes to the study heterogeneity by limiting standardization and adequate patient stratification. A recent expert consensus proposed a Mayo clinical score of at least 4 as a definition of mild to moderate UC including endoscopic activity (at least Mayo 2), rectal bleeding score ≥ 1, and impact on daily clinical activities [62]. Of course, prospective studies are warranted to validate this definition and its widespread use. Secondly, there are several formulations of 5-ASA characterized by different dosages and characteristics. Although no differences in efficacy and safety have been identified, there is no evidence evaluating the efficacy of switching from one type of 5-ASA to another [13,15]. Studies specifically designed to evaluate whether switching to a different 5-ASA can improve medication adherence without affecting patient outcomes are needed. Finally, there are no data on so-called exit strategies for patients with mild to moderate UC. Overall, 5-ASA maintenance therapy is associated with a better prognosis [13]. Approximately 20% of patients treated with 5-ASA achieve disease clearance, a state of profound remission defined as a simultaneous clinical, endoscopic, and histological remission [63,64]. Achieving disease clearance is associated with a reduction in the risk of hospitalization and surgery [65]. However, the impact of discontinuing therapy in patients with long-standing remission is not known and deserves to be further investigated in randomized clinical trials.

## 5. Conclusions

This is the first Middle East expert consensus to provide practical recommendations for managing patients with mild to moderate ulcerative colitis. Through a solid and well-validated Delphi methodology, eight statements have been proposed to standardize the care to UC patients and improve their quality of life. These statements essentially provide structured guidance for healthcare providers in treating and managing patients with mild to moderate UC. They cover induction, maintenance, and adjustment of therapies to achieve optimal disease control and to reduce the risk of complications. These recommendations should be interpreted and applied in clinical practice according to individual patient needs.

## Figures and Tables

**Table 1:** Approved statements.

**Statement 1**	*We suggest the use of oral 5-ASA (from 2.0 to 4.8 g/day) combined with topical 5-aminosalicylates for 8 weeks to induce remission in patients with mild to moderate proctitis (suppository 1 g/day) or left-sided ulcerative colitis/pancolitis (enema ≥ 1 g/day).*	agreement 100%
**Statement 2**	*We recommend to optimize oral 5-aminosalicylates (≥4 g/day) in patients with mild to moderate ulcerative colitis who do not respond to therapy with oral 5-aminosalicylates at a dose < 4 g/day or experience clinical, biochemical, or endoscopic recurrence of disease.*	agreement 100%
**Statement 3**	*5-aminosalicylates at a dose ≥4 g/day are not associated with an increased risk of adverse events or nephrological damage compared with 5-aminosalicylates at a dose ≤2 g/day.*	agreement 100%
**Statement 4**	*In case of loss of response upon de-escalation of medical therapy, 5-aminosalicylates should be re-escalated (≥ 4 g/day) and maintained at stable dosage.*	agreement 87.5%
**Statement 5**	*In patients with mild to moderate ulcerative colitis who are primarily unresponsive or losing response to optimized dose of 5-aminosalicylates, budesonide MMX (9 mg/day) could be considered as add-on therapy for 8 weeks to induce disease remission.*	agreement 100%
**Statement 6**	*If there is no clinical response to therapy within 4 weeks, medical therapy should be escalated in patients with mild to moderate ulcerative colitis.*	agreement 93.7%
**Statement 7**	*Oral 5-aminosalicylates at a dose ≥ 2 g/day are recommended to maintain disease remission and prevent colorectal cancer risk in mild to moderate ulcerative colitis.*	agreement 100%
**Statement 8**	*Oral 5-aminosalicylates should be administered once a day to improve adherence in patients with ulcerative colitis.*	agreement 100%

## Data Availability

No new data were generated or analyzed in support of this research.

## Supplementary Materials

The following supporting information can be downloaded at: https://www.mdpi.com/article/10.3390/jcm12216929/s1, Supplementary Table S1: List of the statements preliminary proposed.
