# Supplementary material for: Treatment of Patients with Mild to Moderate Ulcerative Colitis: A Middle East Expert Consensus
Source: J Clin Med. 2023 Nov 4;12(21):6929. doi: 10.3390/jcm12216929 (PMC10650478; doi:10.3390/jcm12216929)
Supplement: Supplementary file 1 [file jcm-12-06929-s001.zip › jcm-2675981-supplementary.pdf]

Supplementary Materials

Table S1. List of the statements preliminary proposed.

**Statement 1**	**We suggest the use of oral 5-ASA (from 2,0 to 4,8g/day) combined with rectal 5-aminosalicylates for 8 weeks to induce remission in patients with mild to moderate proctitis (suppository 1 g/day) or left-sided ulcerative colitis/pancolitis (enema ≥ 1g/day).**
**Statement 2**	We recommend to optimize oral 5-aminosalicylates (≥ 4g/day) in patients with mild to moderate ulcerative colitis who do not respond to therapy with oral 5-aminosalicylates at a dose < 4g/day or experience clinical, biochemical, or endoscopic recurrence of disease.
**Statement 3**	5-aminosalicylates at a dose ≥4g/day are not associated with an increased risk of adverse events or nephrological damage compared with 5-aminosalicylates at a dose ≤2g/day.
**Statement 4**	Optimized oral 5-aminosalicylates should be de-escalated (≥ 2g/day) after 8 weeks of therapy.
**Statement 5**	In case of loss of response upon de-escalation of medical therapy, 5-aminosalicylates should be re-escalated (≥ 4g/day) and maintained at stable dosage.
**Statement 6**	In patients with mild to moderate ulcerative colitis who are primarily unresponsive or losing response to optimized dose of 5-aminosalicylates, budesonide MMX (9mg/day) could be considered as add-on therapy for 8 weeks to induce disease remission.
**Statement 7**	If there is no clinical response to therapy within 4 weeks, medical therapy should be escalated in patients with mild to moderate ulcerative colitis.
**Statement 8**	Oral 5-aminosalicylates at a dose ≥ 2 g/day are recommended to maintain disease remission and prevent colorectal cancer risk in mild to moderate ulcerative colitis.
